# Dolutegravir Inhibition of Matrix Metalloproteinases Affects Mouse Neurodevelopment

**DOI:** 10.1007/s12035-021-02508-5

**Published:** 2021-08-14

**Authors:** Aditya N. Bade, JoEllyn M. McMillan, Yutong Liu, Benson J. Edagwa, Howard E. Gendelman

**Affiliations:** 1 Department of Pharmacology and Experimental Neuroscience, University of Nebraska Medical Center, Omaha, NE 68198-5800 USA; 2 Department of Radiology, University of Nebraska Medical Center, Omaha, NE 68198 USA; 3 Department of Pharmaceutical Sciences, University of Nebraska Medical Center, Omaha, NE 68198 USA

**Keywords:** Dolutegravir, HIV-1, Antiretroviral drug, Pregnancy outcomes, Matrix metalloproteinases, Neurodevelopment

## Abstract

Dolutegravir (DTG) is a first-line antiretroviral drug (ARV) used in combination therapy for the treatment of human immunodeficiency virus type-1 (HIV-1) infection. The drug is effective, safe, and well tolerated. Nonetheless, concerns have recently emerged for its usage in pregnant women or those of child-bearing age. Notably, DTG-based ARV regimens have been linked to birth defects seen as a consequence of periconceptional usages. To this end, uncovering an underlying mechanism for DTG-associated adverse fetal development outcomes has gained clinical and basic research interest. We now report that DTG inhibits matrix metalloproteinases (MMPs) activities that could affect fetal neurodevelopment. DTG is a broad-spectrum MMPs inhibitor and binds to Zn^++^ at the enzyme’s catalytic domain. Studies performed in pregnant mice show that DTG readily reaches the fetal central nervous system during gestation and inhibits MMP activity. Postnatal screenings of brain health in mice pups identified neuroinflammation and neuronal impairment. These abnormalities persist as a consequence of in utero DTG exposure. We conclude that DTG inhibition of MMPs activities during gestation has the potential to affect prenatal and postnatal neurodevelopment.

**Supplementary Information:**

The online version contains supplementary material available at 10.1007/s12035-021-02508-5.

## Introduction

The World Health Organization updated guidance recommends continuation or initiation of antiretroviral therapy (ART) in all pregnant and breastfeeding women living with human immunodeficiency virus type-1 (HIV-1) infection [1]. Around 1.3 million HIV-1-infected pregnant women worldwide give birth each year [2]. With increased numbers of infected pregnant women receiving ART, over a million HIV-1-exposed but uninfected (HEU) children are born each year [2, 3]. This results in potential exposures of fetuses to antiretroviral drugs (ARVs) during development. Importantly, emerging data demonstrates that HEU children have inferior health outcomes compared to their HIV-1-unexposed counterparts [4]. This highlights an immediate need to identify any ARV or class thereof associated with adverse pregnancy outcomes. There is a need to define any altered prenatal and postnatal developmental outcomes after ARV exposures and elucidate underlying mechanisms.

Dolutegravir (DTG) is currently recommended as a part of first, second, or salvage regimens for HIV-1-infected patients, in both resource-rich and resource-limited countries (RRCs and RLCs) [1, 5]. This is due to its high potency and genetic barriers to drug resistance [6–8]. Nonetheless, in recent years, concerns for the usage of DTG-based regimens by pregnant women or women of child-bearing age emerged based on apparent increases in fetal abnormalities [2, 9, 10]. In 2018, the interim analysis of the Tsepamo birth surveillance study in Botswana reported neural tube defects (NTDs) at eight times higher rates in babies born to mothers who received DTG periconceptionally compared to those who had taken other ARVs [9]. Extended surveillance in Botswana showed lower risk of NTDs, yet the risk was 2–3 times higher than other ARTs at conception [10]. In addition, DTG-associated fetal defects were reported when pregnant mice were given the drug at therapeutic levels [11]. Both clinical and preclinical studies observed the risk of NTD’s following in utero DTG exposure and stressed the importance of identifying unknown potential adverse drug effects (ADEs) [9–11]. These include any injuries resulting from in utero DTG exposures, including biological, physiological, metabolic, or functional harm to the fetal central nervous system (CNS), particularly in babies born without structural, brain or spinal cord, malformations [12, 13]. Recently, the Surveillance Monitoring for ART Toxicities (SMARTT), an observational study, reported associations between in utero DTG exposures and neurologic abnormalities in newborns and during development [2]. These studies underscored the importance for identifying both the neurologic abnormalities and the mechanisms for how they could occur following in utero DTG exposure.

DTG is an integrase strand transfer inhibitor (INSTI) that blocks the action of the viral integrase enzyme, which is responsible for the insertion of the viral genome into the host cellular DNA. It possesses a metal-binding pharmacophore (MBP, also referred as a metal-binding group, MBG) for engagement with active metal ion (Mg^++^) sites in the HIV-1 integrase [14]. With a metal chelating motif in the chemical structure, DTG possesses potential to interact with other metalloenzymes that are critical for normal biological activities, including cell proliferation, differentiation, signaling, nucleic acid modification, and protein degradation. Out of all metalloenzymes, matrix metalloproteinases (MMPs) are a class of Zn^++^-dependent enzymes that are essential during CNS developmental processes such as axonal growth and guidance, synaptic development and plasticity, myelinogenesis, and angiogenesis [15–26]. Dysregulation of MMPs can cause detrimental effect on neurodevelopment and normal functioning. Thus, we hypothesized that DTG inhibition of MMPs activities by chelating Zn^++^ at the catalytic domain during gestation can certainly affect neurodevelopment.

To each of these ends, the findings contained in the current report demonstrate, for the first time, a novel, yet, previously undisclosed pathway for DTG-associated developmental neurotoxicity that follows in utero exposure in mice. The identified pathway was linked to DTG inhibition of MMPs activities. Interestingly, DTG was found to be a broad-spectrum inhibitor of MMPs. In mice, DTG exposure to the developing embryo CNS during gestation inhibited MMPs activities. Further postnatal evaluation of brain health in mice pups following DTG exposures identified neuroinflammation and consequent neuronal damage. These data demonstrate an association between DTG dysregulation of MMPs activity during gestation and consequent neurotoxicity. We conclude that DTG inhibition of MMPs activities during gestation can affect neurodevelopment in mice warranting continued observations in humans.

## Methods

### Gelatin Zymography

The gelatin zymography assay was performed to determine MMP-9 and MMP-2 activity following treatment of THP-1 cells with DTG. THP-1 cells were plated in 12-well plates at a density of 1 × 10^6^ cells per well. First, THP-1 were treated with phorbol-12-myristate-13-acetate (PMA) for 24 h to promote the differentiation of cells into macrophages and stimulate MMP secretion. Following 24-h treatment with PMA, cells were treated with DTG (1, 10, or 100 µM) or vehicle (control) for 18 h. For each treatment, biological triplicate samples were utilized. Following treatment, cell medium was collected from each well and centrifuged at 800 × g for 10 min at 4 °C to remove any cell debris. Collected samples were stored at − 80 °C for further analysis. A 10% SDS–polyacrylamide gel containing 0.1% gelatin was loaded with 3 µg of protein from cell medium for gelatin zymography. In parallel, second 10% SDS–polyacrylamide gel was used to assess equal protein loading. In addition, recombinant human MMP-2 and MMP-9 (10 ng each) were loaded as standards (EMD Millipore, Burlington, MA). Gel ran at 60 V until the loading dye reached the bottom of the gel. After the run, the gelatin gel was washed with water for 15 min and incubated in renaturation buffer [2.5% (v/v) Triton X-100 in Milli-Q water] for 60 min at room temperature. The renaturation buffer was replaced with a fresh renaturation buffer at the interval of 30 min. Later, the gel was incubated in developing buffer (50 mM Tris–HCl, pH 7.5, 5 mM CaCl_2_, 0.2 M NaCl, and 0.02% Brij-35) for 48 h at 37 °C in an incubator shaker (Innova 42, New Brunswick Scientific, Edison, NJ). The gel was later washed with water for 15 min and then stained with 0.2% Coomassie Brilliant Blue R-250 (BIO-RAD, Hercules, CA) for 1 h. After staining, the gel was washed with water for 15 min followed by washing with destaining solution (BIO-RAD, Hercules, CA) for 30 min. The gel was washed one more time for 15 min with water to remove any distaining solution. Finally, the stained gel was imaged by iBright 750 Imaging Systems (Invitrogen, Carlsbad, CA). Relative activity of MMPs was measured by quantitating band density by ImageJ software and normalized with respective standard.

### Cytotoxicity Measurements

Cellular viability following treatment with DTG was evaluated by 3-(4,5-dimethylthiazol-2-yl)-2,5-diphenyltetrazolium bromide (MTT) assay. THP-1 cells were plated in 96-well plates at a density of 0.08 × 10^6^ cells per well. Cells were treated with 1, 10, or 100 µM DTG for 18 h in presence of PMA to determine the cellular toxicity. Vehicle-treated cells were used as controls. For each treatment, twelve samples were utilized. Cells were washed with PBS and incubated with 100 µL/well of MTT solution (5 mg/mL) for 45 min at 37 °C. After incubation, MTT solution was removed, and DMSO (200 µL) was added to each well. Absorbance was measured at 490 nm on a Molecular Devices SpectraMax M3 plate reader with SoftMax Pro 6.2 software (Sunnyvale, CA). Absorbance was compared to that of control cells to determine cytotoxicity.

### Fluorometric Substrate Assays

MMP inhibitor profiling kit from Enzo life sciences (BML-AK308, Farmingdale, NY) was utilized to assess quantitative inhibition profile of DTG against ten different MMPs. Kit contained a panel of ten MMPs and a quenched fluorogenic substrate OMNIMMP® RED. Composition of a quenched fluorogenic substrate was TQ3-GABA-Pro-Cha-Abu-Smc-His-Ala-Dab(6’-TAMRA)-Ala-Lys-NH2 [TQ3 = quencher; GABA = 4-aminobutyric acid; Cha = L-cyclohexylalanine; Abu = 2-aminobutyric acid; Smc = S-methyl-L-cysteine; Dab = 2,4-diaminobutyric acid; 6’-TAMRA = 6’-tetramethylrhodamine]. Experiment was performed as per instructions provided in the manufacturer’s manual. The assays were performed in a 96-well, black, flat, microplate format. The compound NNGH was provided as a prototypic control inhibitor. Briefly, DTG at five different concentrations (0.1, 1, 10, 100, and 1000 µM) and NNGH were incubated with each MMP at 37 °C for 60 min. Untreated MMPs were used as control. The experiment was performed in biological triplicates. After 60-min incubation, fluorogenic substrate OMNIMMP® RED was added to each reaction/well to start the reaction. The plate was continuously read at Ex/Em = 545/576 nm (cutoff at 570 nm) for 30 min with 1-min time intervals at constant temperature, 37 °C, using Molecular Devices SpectraMax M3 plate reader with SoftMax Pro 6.2 software. The half maximal inhibitory concentration (IC_50_) value of DTG for each enzyme was calculated using four-parameter curve fit using R software package.

### Molecular Docking

Homology models of MMP-8, MMP-9, MMP-14, and MMP-19 were generated on a template of MMP-2 (PDB ID: 1HOV) using the Homology Modeling module of the YASARA Structure [27] program package. The models of MMP-9, MMP-14, and MMP-19 have *Z*-scores less than − 2.0 and were subjected to 500 ns molecular dynamics simulations to refine their structures. The Schrodinger software suite release 2020–4 (New York, NY) was used for all molecular dynamics simulations and molecular docking calculations. All molecules were parametrized using the OPLS3e [28] force field. Each homology model was placed in an orthorhombic box of TIP4P water with periodic boundaries at least 10 Å from any solute molecule. The simulation cells were neutralized with the addition of Na^+^ or Cl^−^ ions as appropriate. The systems were relaxed following the standard protocol as implemented in the Schrodinger suite. Production molecular dynamics were run for 500 ns with default settings. The representative structure of the largest cluster from each simulation was chosen for docking calculations. Induced-fit binding as implemented in Schrodinger was used with default settings, except that the high-accuracy XP mode was chosen for the Glide docking steps. All ranked poses were required to have at least one bond with the active site zinc ion; other poses were not considered.

### Animals

For all animal experiments, C3H/HeJ (10–12 weeks of age, male and female) were purchased from the Jackson Laboratory (Bar Harbor, ME), and were housed in microisolator cages containing autoclaved corncob bedding, and maintained on a 12‐h light‐dark cycle in climate‐controlled laboratory animal facilities approved by the University of Nebraska Medical Center Institutional Animal Care and Use Committee (IACUC). Mice were given free access to irradiated rodent feed (TD. 180,911, Envigo Teklad diet, Madison, WI) and sterile water. Animals were acclimated to the environment for approximately 7–14 days to determine that they were healthy based on observed behavior and weight gain. A female was placed with a male overnight for the purpose of timed matings. The following morning males were removed from the cages and positive vaginal plugs were identified. Females exhibiting vaginal plugs were weighed and were randomly distributed to treatment (DTG) or control group. Male mice did not receive any drug/chemical exposure. The time of conception was considered to be midnight on the evening of the mating. Treatment of plugged females was started at the morning of the day of plug detection (gestation day (GD) 0.5).

### PK and BD of DTG During Pregnancy

Pharmacokinetic (PK) and biodistribution (BD) profiles of DTG were determined in pregnant female C3H/HeJ mice (10–12 weeks old). Pregnant mice were administered DTG daily at a dose of 50 mg/kg. DTG solution in dimethylsulfoxide:Solutol®:50 mM N-methylglucamine in 3% mannitol (1:1:8, v:w:v) was administered by oral gavage throughout the entire gestational period. Drug administration was started at the day of plug detection (GD 0.5) and continued up to the day of birth of pups. Blood samples were collected from dams into heparinized tubes by cheek puncture (submandibular vein) using a 5-mm lancet (MEDIpoint, Inc., Mineola, NY) at GD 8.5 and 16.5 and postnatal days (PND) 4, 12, and 21 for plasma collection. Collected blood samples were centrifuged at 2000 × g for 8 min for plasma collection and quantitation of plasma DTG concentrations. DTG concentrations were also quantitated in harvested placental tissue at GD 16.5. In addition, biodistribution of DTG to fetal brain tissue was determined at GD 16.5 and PND 4. DTG levels were quantitated in plasma, and tissue homogenates by ultraperformance liquid chromatography-tandem mass spectrometry (UPLC-MS/MS) according to previously published protocols [29]. Each sample (plasma or tissue) evaluated for DTG levels was randomly selected from distinct litters.

### Embryo Phenotype Assessments

Effect of DTG exposure during gestation on embryo development was evaluated. Pregnant C3H/HeJ mice were administered daily with DTG solution at a dose of 50 mg/kg or vehicle [dimethylsulfoxide:Solutol®:50 mM N-methylglucamine in 3% mannitol (1:1:8, v:w:v)] by oral gavage. DTG or vehicle was administered by oral gavage from GD 0.5 to GD 15.5. Pregnant dams were randomly assigned to DTG or vehicle (control) group. Weight gain in dams was measured on GD 0.5, 5.5, 10.5, and 15.5. At GD 16.5, dams were humanely euthanized, and embryos and placenta were harvested. Embryos were evaluated for NTDs. At the time of harvest, the total number of implants (litter size, resorption rates, or viable embryos) was recorded for each pregnancy. Further, whole brain tissues from normal viable embryos were isolated and processed for examination of MMP expression and activity.

### MMP Activity Measurements in Embryonic Brain

Broad-spectrum MMP activity was measured in whole brain tissue homogenates of normal, viable embryos (GD 16.5) using a quenched fluorogenic substrate, OMNIMMP® RED (BML-P277-0100, Enzo Life Sciences). Whole brain tissues were isolated from randomly selected embryos from different dams for both DTG and control groups. Tissues were homogenized in NP-40 lysis buffer using a Qiagen TissueLyzer II (Valencia, CA). Tissue lysates were centrifuged at 16,000 g at 4 °C for 20 min and supernatants were collected. Total protein levels in each sample were quantitated using Pierce™ BCA Protein Assay Kit (Thermo Fisher Scientific, Waltham, MA). The assays were performed in a 96-well, black, flat, microplate format. Fifty micrograms of protein for each sample was incubated with buffer (BML-KI175-0020, Enzo Life Sciences) at 37 °C for 60 min. Each sample was platted in duplicates. Later, reaction was started by adding fluorogenic substrate. Plate was read at similar instructions mentioned above for fluorometric substrate assay. Ilomastat (GM6001; broad-spectrum MMP inhibitor, ab120845, abcam) was added to randomly selected protein samples in additional wells at 20 µM concentration as an assay control.

### MMP Expression

At GD 16.5, embryo whole brain tissues were isolated and homogenized in lysis buffer. Whole brain tissue homogenates were analyzed for expression of MMP-2, 9, and 14 using Western blot assay. Six pups were randomly selected from 6 different dams for both DTG- and vehicle-treated groups. Tissue lysates were centrifuged at 16,000 × g at 4 °C for 20 min and supernatants were collected. Total protein levels in each sample were quantitated using Pierce™ BCA Protein Assay Kit (Thermo Fisher Scientific). Fifteen micrograms of protein for each sample was ran on 10% SDS–polyacrylamide gel. Proteins were transferred to activated PVDF membrane. Membranes were blocked with 5% non-fat milk (BIO-RAD) for 1 h and incubated with individual primary antibody overnight at 4 °C on a shaker. The primary antibodies used were rabbit monoclonal anti-MMP-2 (ab181286, abcam, Cambridge, MA), rabbit polyclonal anti-MMP-9 (ab38898, abcam), rabbit monoclonal anti-MMP-14 (ab51074, abcam), rabbit monoclonal GAPDH (Cell Signaling Technology, Inc., Danvers, MA), and mouse monoclonal β-actin (Santa Cruz Biotechnology, Dallas, TX). All the primary antibodies were used at 1:2000 dilution. After overnight incubation with primary antibody, the membranes were washed with Tris-buffered saline with 0.1% Tween® 20 detergent (TBST) three times (5 min each wash). Membranes were then incubated with secondary antibody for 1 h at room temperature. The secondary antibodies used were goat anti-rabbit IgG H&L (HRP) (ab205718, abcam) and goat anti-mouse IgG H&L (HRP) (ab205719, abcam). For secondary antibodies, 1:10,000 dilutions were used. Blots were developed using SuperSignal™ West Femto Maximum Sensitivity Substrate (Thermo Fisher Scientific) and iBright 750 Imaging System. Band intensity was analyzed using ImageJ software.

### Diffusion Tensor Imaging

In vivo bioimaging (diffusion tensor imaging (DTI) and magnetic resonance spectroscopy (^1^H MRS)) was performed using a 7 Tesla/16 cm Bruker PharmaScan or a 7 Tesla 21 cm Bruker Biospec (Karlsure, Germany) MRI/MRS system. At PND 28–34, DTI was performed on live mice pups to determine microstructural changes. Animals from both DTG- and vehicle-treated (control) groups were scanned. Pups (male and female) were randomly selected from each dam. As described in our previous work [30], a 4-segment echo planar imaging (EPI) readout with a balanced, rotationally invariant and alternating polarity icosahedral scheme (12 directions) and *b* value = 800 s/mm^2^ were utilized. Diffusion Toolkit (http://trackvis.org/dtk/) was used to calculate DTI metrics, fractional anisotropy (FA). Region of interest (ROI) analysis was performed to measure DTI metrics on hippocampus (HI), cortex (CT), striatum (ST), thalamus (TH), hypothalamus (HY), and cerebellum (CE).

### Magnetic Resonance Spectroscopy

After DTI measurements, the same animals were scanned by ^1^H MRS to measure metabolite concentrations. *N*-Acetylaspartate (NAA), total choline (tCho), and total creatine (tCre) were measured in HI region. Animals from both groups, DTG and control, were scanned. SemiLASER localization with timing parameters (TE/TR = 40/4000 ms, 576 averages, 2048 points) was used to obtain MRS data [31]. Voxel was located on HI. All first- and second-order shim terms were automatically adjusted using MAPSHIM® (Bruker, Billerica, MA). A final shim performed manually, if necessary, to achieve a water line width of 10–15 Hz. The water signal was suppressed by variable power radiofrequency (RF) pulses with optimized relaxation delays (VAPOR) [32]. MRS data were quantified using LCModel (LCMODEL Inc., CA). Results were expressed as a percentage of the sum of all 3 metabolites as a semi-quantitative method for reporting metabolite concentrations in institutional units (I.U.). Glycerophosphocholine and phosphocholine were added and reported as total choline-containing compounds.

### Next-Generation Sequencing

After MRI imaging, animals were humanely euthanized and brain regions were isolated. Brain tissues were flash frozen in liquid nitrogen and stored in − 80 °C. Tissue RNA was isolated from HI region using RNeasy Mini Kit (QIAGEN, Germantown, MD). HI region from 4 randomly selected pups from 4 different dams for each group (DTG-treated and vehicle-treated) was used to isolate RNA. Nucleic acid integrity was assessed, and the RNA samples were deep sequenced using 100 bp/read and ≤ 40 million reads/sample using an Illumina HiSeq 2500 Sequence Analyzer (Illumina, Inc., San Diego, CA, USA). The original fastq format reads were merged and trimmed using the fqtrim tool (https://ccb.jhu.edu/software/fqtrim) to remove adapters, terminal unknown bases (Ns), and low-quality 3′ regions (Phred score < 30). Quality was assessed for each trimmed file with FASTQC for high-throughput sequence data. The trimmed fastq files were processed utilizing STAR [33] as the aligner and RSEM [34] as the tool for annotation and quantification at both gene and isoform levels. The trimmed fastq files were mapped to the mouse reference genome according to the corresponding sample species. The gene and transcript abundance were measured as transcripts per kilobase million (TPM) values. TPM was calculated by normalizing the gene length, followed by the sequencing depth to make easier comparison of the proportion of reads that mapped to a gene in each sample. The identified set of genes was then used to examine the differential expression of various genes between the sample groups in the R statistical software environment with packages from Bioconductor and to identify pathways using Ingenuity pathway analysis (https://www.qiagen-bioinformatics.com). Comparison analysis was performed between DTG-treated and vehicle-treated (control) samples. We identified differentially expressed genes and associated pathways with log_2_ (fold change) < 0.5 and >  − 0.5 and *p* values < 0.05. The top ranking upregulated and downregulated genes were selected to plot the graphs.

### Statistical Analysis

Statistical analyses were conducted using GraphPad Prism 7.0 software (La Jolla, CA). Data from in vitro studies were expressed as mean ± standard error of the mean (SEM) with a minimum of 3 biological replicates. Results from in vivo studies were expressed as mean ± SEM with a minimum of 4 biological replicates. For comparisons between two groups, *t* test (two-tailed) with Welch’s correction was employed. A one-way ANOVA followed by Dunnett’s test was used to compare three or more groups. Statistical significances were denoted as follows: ^#^*p* < 0.1, *p < 0.05, **p < 0.01, ***p < 0.001, ****p < 0.0001.

### Study Approvals

All animal studies were approved by the University of Nebraska Medical Center Institutional Animal Care and Use Committee (IACUC) in accordance with the standards incorporated in the Guide for the Care and Use of Laboratory Animals (National Research Council of the National Academies, 2011).

## Results

### DTG Is a Broad-Spectrum MMPs Inhibitor

**Fig. 1:**
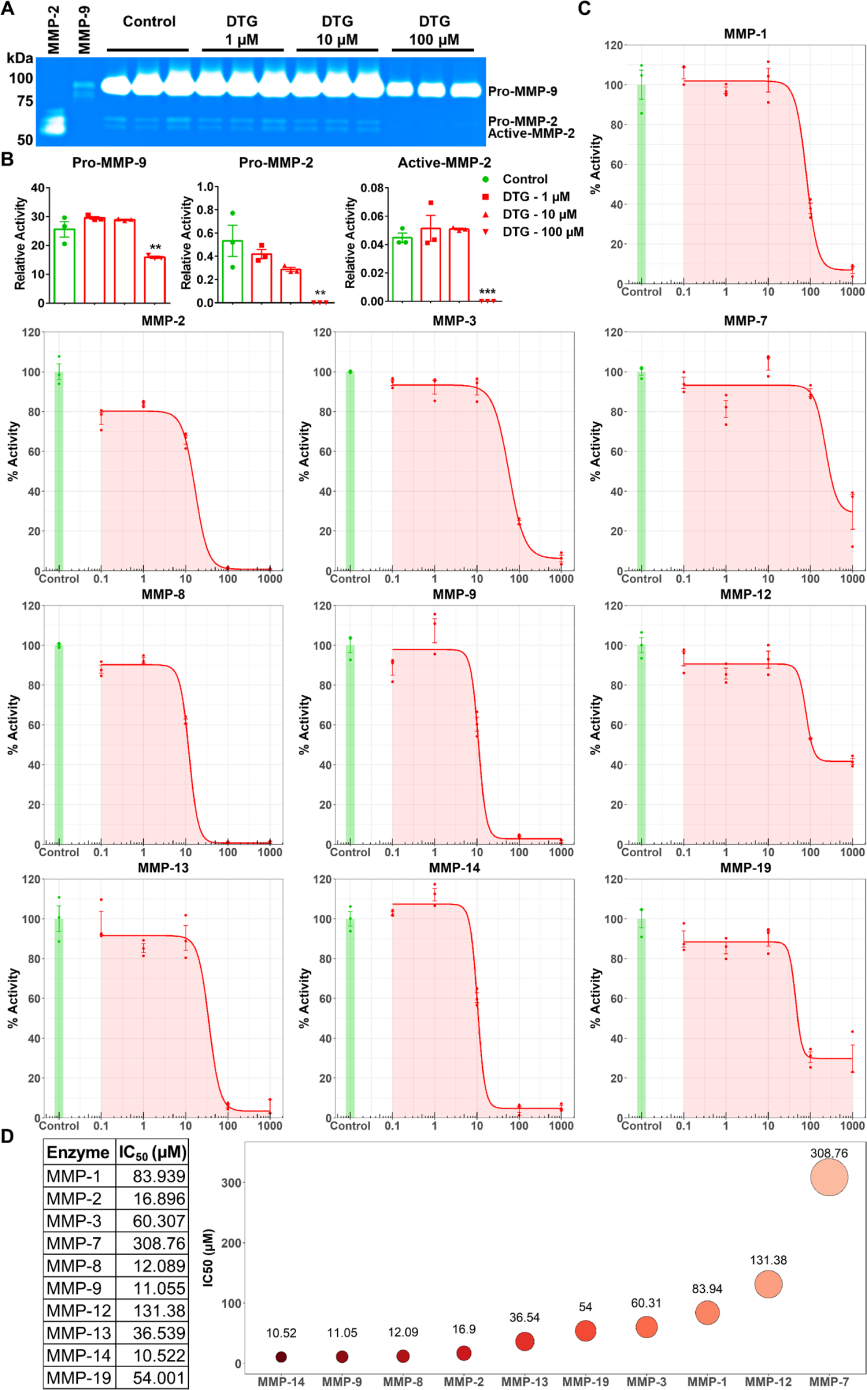
DTG inhibition of MMPs. **A** Gelatin zymogram. Activity of MMP-2 and MMP-9 was evaluated in serum-free medium of THP-1 cells following treatment with DTG (1, 10, or 100 µM). Vehicle-treated cells were used as control. Human recombinant enzymes were used as standards. **B** Relative activity of MMP-9 or MMP-2 was measured and normalized with respective standard. A one-way ANOVA followed by Dunnett’s test was used to compare activity of individual MMP between each treatment concentration and untreated control (**p < 0.01, ***p < 0.001). **C** Fluorometric substrate assay. Inhibition profile of DTG (0.1, 1, 10, 100, and 1000 µM) was determined against ten human recombinant MMP enzymes. **D** IC_50_ values for DTG against each MMP were calculated from dose–response curve (fluorometric substrate assay) using four-parameter hill-slope model. For both assays, data are expressed as the mean ± SEM, *N* = 3 biological replicates. Experiments were repeated independently three times with equivalent results

To affirm that DTG inhibits MMP activity, two commonly used assays to study MMP activity and their inhibitors were performed. These included gelatin zymography and fluorometric substrate assays [35, 36]. For gelatin zymography, cell culture of the monocyte THP-1 cell line was employed. Phorbol-12-myristate-13-acetate (PMA) was used to differentiate THP-1 cells into macrophage-like cells and to induce MMP secretion [37, 38]. Herein, PMA-stimulated THP-1 cells were treated with escalating DTG concentrations (1, 10, or 100 µM) for 18 h in serum-free culture medium. To determine the proteolytic activity of MMPs 2 and 9 (gelatinases), equal amount of protein (3 µg) from cell culture medium was loaded on SDS-PAGE containing gelatin. Gel area digested by both MMPs was visualized with Coomassie blue (Fig. 1A). Pro and active forms of both enzymes were confirmed with respective standards, human recombinant MMP-2 and MMP-9 (lanes 1 and 2; Fig. 1A). Decrease in activity of MMP-2 and 9 was observed following DTG treatment compared to vehicle-treated controls on gelatin zymogram (Fig. 1A). Relative activity of both pro and active forms of MMP-2 and pro form of MMP-9 was significantly decreased by DTG treatment at 100 µM concentration compared to controls (Fig. 1B). Cellular vitality assessed in THP-1 cells by the 3-(4,5-dimethylthiazol-2-yl)-2,5-diphenyltetrazolium bromide (MTT) assay showed no DTG-induced cytotoxicity at 1, 10, or 100 μM drug concentration (Supplementary Fig. 1). After confirmation from the standard gelatin zymography assay that DTG inhibits activity of MMP-2 and MMP-9, a fluorometric substrate assay was performed to assess quantitative inhibition profile of DTG against different MMPs (Fig. 1C). Ten MMPs included in assay can affect embryonic development and represent each of the MMP subfamilies [15–26]. Dose-dependent enzymatic activity inhibition over time was measured. Resulting reaction rates were normalized as percentage of the respective untreated control and plotted against drug concentrations (Fig. 1C). The half maximal inhibitory concentration (IC_50_) for each enzyme was calculated using a four-parameter curve fit (Fig. 1D). DTG inhibited activity of all ten MMP enzymes at different extent. IC_50_ values for DTG inhibition of enzyme’s activity for MMP-2 (IC_50_: 16.89 µM), MMP-8 (IC_50_: 12.08 µM), MMP-9 (IC_50_: 11.05 µM), and MMP-14 (IC_50_: 10.52 µM) (Fig. 1D) were determined. Other IC_50_ values of DTG were 83.93, 60.30, 308.76, 131.37, 36.53, and 54.0 µM for MMP-1, 3, 7, 12, 13, and 19, respectively. To visualize the extent of inhibition among MMPs, the sequence of IC_50_ values for DTG inhibition of MMPs is shown in a bubble chart (Fig. 1D). This chart starts with the lowest IC_50_ value (MMP-14; IC_50_: 10.52 µM; dark red color) and increases to higher IC_50_ values with respective MMPs, which are presented in lighter red color shades (Fig. 1D). These results were further validated in comparison to doxycycline which is the only US Food and Drug Administration (FDA)-approved broad-spectrum MMPs inhibitor [39] (Supplementary Fig. 2). At 20 µM concentration, DTG had higher inhibitory effect on MMP-2, 8, 9, 13, and 14 compared to doxycycline. Altogether, gelatin zymography and fluorometric substrate assays demonstrated that DTG is a broad-spectrum MMPs inhibitor.

### DTG Binds to Zn^++^ at Catalytic Domain of MMP to Inhibit the Enzyme’s Activity

**Fig. 2:**
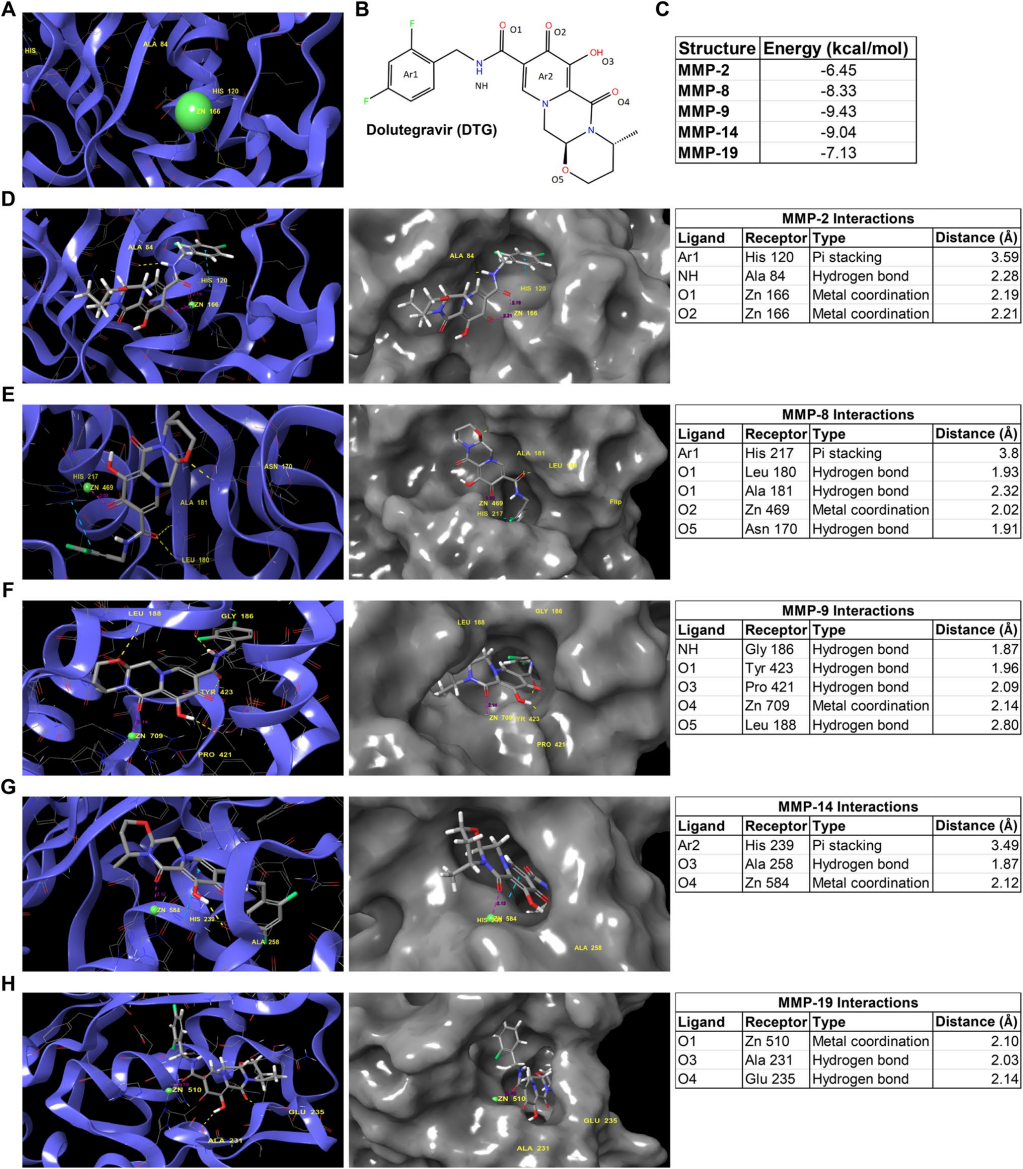
Molecular interaction between DTG and MMP. **A** A 3D representative image of MMP-2 catalytic domain containing Zn^++^ (green ball) is shown. **B** Chemical structure of DTG with ligand labeling used for molecular docking. **C** Calculated binding energies for each MMP with DTG using Schrodinger’s software suite. **D**–**H** 3D representations of molecular docking complex of DTG with **D** MMP-2, **E** MMP-8, **F** MMP-9, **G** MMP-14, and **H** MMP-19. 3D representations of molecular docking complexes are shown in ribbon (blue color; left side of the figure) and surface interaction (gray color; middle of the figure) formats. Interactions with Zn^++^ (green ball) and other amino acids (yellow color) are identified. Interactions with Zn^++^ are shown by pink dotted line. Hydrogen bond interaction with amino acids is shown by yellow dotted line. Pi staking is shown by blue dotted line. Interaction details for each complex are provided in the tabular format (right side of the figure)

With the establishment that DTG inhibits MMP activity, computational molecular docking evaluations were completed using Schrodinger’s software. These tests were performed to identify the mechanism through which DTG interacts with catalytic domain of MMPs. MMPs are part of a family of structurally related Zn^++^-dependent endopeptidases [39]. As DTG possesses a prominent MBP for binding to the metal ions [14], we hypothesized that DTG inhibits activity of MMP by binding to Zn^++^ at the catalytic domain. Since DTG inhibits MMP-2, 8, 9, 14, and 19 to a higher extent (low IC_50_ values, Fig. 1C, D), these enzymes were used for DTG-MMP mechanistic studies. Herein, induced fit docking used a combination of the Glide and Prime programs in the Schrodinger suite. All docking scores used the highest accuracy Glide XP mode. A representative 3D image of MMP-2 catalytic domain with Zn^++^ ion (bright green ball) is shown in Fig. 2A. Chemical structures of DTG with ligand interaction sites (numbered and colored) are shown in Fig. 2B. Docking simulation of DTG into individual MMP showed highest binding energy of − 6.45, − 8.33, − 9.43, − 9.04, or − 7.13 kcal/mol for MMP-2, 8, 9, 14, or 19, respectively (Fig. 2C). DTG formed a metal coordination complex with Zn^++^ in the catalytic domain of all tested MMPs (Fig. 2D–H). Metal coordination with Zn^++^ occurred at Zn 166, 469, 709, 584, and 510 of MMP-2, 8, 9, 14, and 19 respectively (Fig. 2D–H). Other interactions that occurred were hydrogen bonding and pi stacking. Specifically, pi stacking occurred at histidine 120 amino acid of MMP-2, histidine 217 amino acid of MMP-8, and histidine 239 amino acid of MMP-14 (Fig. 2D–H). The hydrogen bond interactions occurred at alanine 84 amino acid of MMP-2; the three hydrogen bond interactions occurred at leucine 180, alanine 181, and asparagine 170 amino acid residues of MMP-8; the four hydrogen bond interactions occurred at glycine 186, tyrosine 423, proline 421, and leucine 188 amino acid residues of MMP-9; the hydrogen bond interactions occurred at alanine 258 amino acid of MMP-14, and the two hydrogen bond interactions occurred at alanine 231 and glutamate 235 amino acid residues of MMP-19 (Fig. 2D–H). The distances of all the enzyme-ligand bonds are shown in the respective enzyme interaction tables (Fig. 2D–H). Overall, the binding energies from the docking simulation validated IC_50_ values (Fig. 1C, D). Computational molecular docking assessments confirmed that DTG is a broad-spectrum inhibitor, and it inhibits MMPs activities by chelating Zn^++^ at the catalytic domain.

Further, we completed preliminary molecular docking assessments for other drugs of INSTIs to determine whether interaction with the Zn^++^ at catalytic domain of MMPs is a class effect. We assessed molecular docking of cabotegravir (CAB), bictegravir (BIC), and raltegravir (RAL) on MMP-2 and 14. Notably, all three INSTIs (CAB, BIC, and RAL) formed receptor interactions with Zn^++^ in the catalytic domain of both MMPs (Supplementary Fig. 3). RAL formed two metal and one cation pi interactions with Zn^++^ at Zn 166 MMP-2 and formed one metal and one cation pi interactions at Zn 584 of MMP-14 (Supplementary Fig. 3A). BIC formed two metal and one salt bridge interactions with Zn^++^ at Zn 166 MMP-2 and formed two metal and one salt bridge interactions at Zn 584 of MMP-14 (Supplementary Fig. 3B). CAB, also, formed two metal and one salt bridge interactions with Zn^++^ at Zn 166 MMP-2 and formed two metal and one salt bridge interactions at Zn 584 of MMP-14 (Supplementary Fig. 3C). The distances of all the enzyme-ligand bonds are shown in the respective enzyme-drug interactions’ table (Supplementary Fig. 3A-3C). Docking simulation of BIC and RAL showed the highest binding energy of − 10.97 kcal/mol for MMP-2 and − 14.53 kcal/mol for MMP-14, and − 6.29 kcal/mol for MMP-2 and − 8.66 kcal/mol for MMP-14, respectively (Supplementary Fig. 3A and 3B). Docking simulation of CAB into MMP-2 and 14 showed the highest binding energy − 8.58 and − 12.98 kcal/mol, respectively (Supplementary Fig. 3C). These molecular docking assessments confirmed that all INSTIs possess chemical abilities for broad-spectrum MMPs inhibition.

### Biodistribution of DTG to Developing Brain During Gestation

**Fig. 3:**
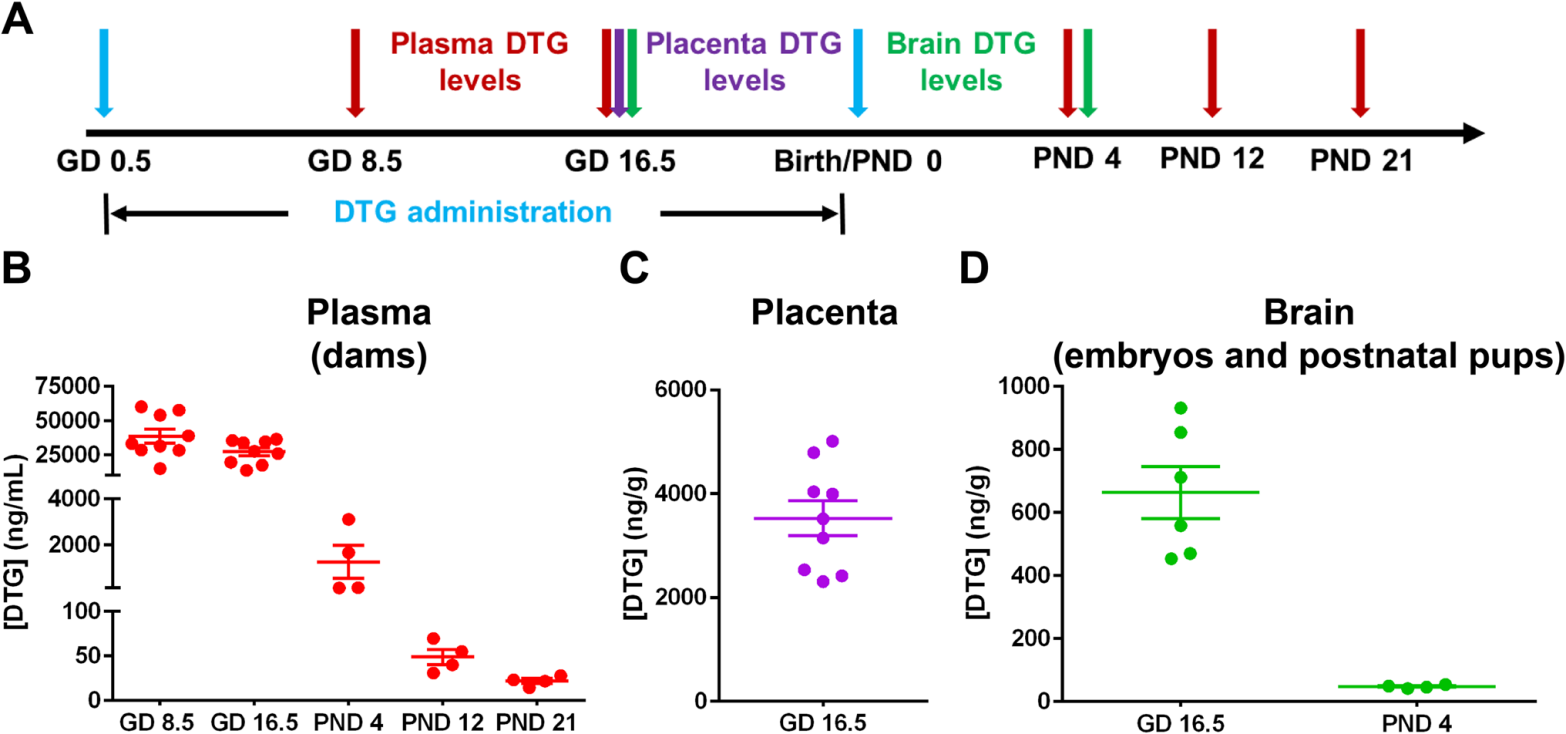
DTG PK and BD during and after pregnancy. **A** Schematic presentation of the experimental timeline. **B** DTG concentrations in plasma of dams. DTG levels were measured during prenatal and postnatal period to evaluate DTG levels in the mother’s blood. **C** DTG concentrations in placental tissue at GD 16.5. **D** DTG concentrations in brain tissue of embryos or pups. Whole brains from embryos and pups were processed for DTG levels at GD 16.5 and after birth at PND 4. **B**–**D** Each sample (plasma or tissue) represents distinct litter. For drug concentration quantitation in all samples, data are expressed as mean ± SEM, *N* = minimum 4 animals at each time point

To assess pharmacokinetic (PK) and biodistribution (BD) profiles of DTG during pregnancy, female C3H/HeJ mice were administered with 50 mg/kg DTG daily by oral gavage. DTG was administered throughout the entire gestational period, beginning at the day of vaginal plug detection [gestation day (GD) 0.5] and up to the day of birth of pups (Fig. 3A). DTG levels were measured in maternal plasma during prenatal and postnatal period, up to postnatal day (PND) 21. As DTG is known to transfer to infants through milk in addition to transplacental transfer [40–42], maternal plasma drug levels were measured up to the day of weaning of pups (PND 21). At GD 8.5 and 16.5, high DTG concentrations were detected in plasma of dams, 38,628.63 ± 4855.53 and 27,296.3 ± 2701.15 ng/mL, respectively (Fig. 3B). Since DTG administration was stopped at the day of birth of pups, plasma DTG levels declined over time to 1244.2 ± 623.20, 48.95 ± 7.39, and 21.97 ± 2.42 ng/mL at PND 4, 12, and 21, respectively (Fig. 3B). In addition, DTG levels were measured in placenta at GD 16.5 with an average tissue concentration of 3528.04 ± 316.56 ng/g (Fig. 3C). The ratio of placental DTG levels to mother’s plasma DTG levels at GD 16.5 was 0.12. Daily DTG administration elicited high DTG levels in embryonic brain tissue (663.6 ± 75.13 ng/g) at GD 16.5 (Fig. 3D). DTG levels were lower in brain tissue of pups at PND 4 (48.1 ± 2.35 ng/g at PND 4) compared to those in embryonic brain tissue (Fig. 3D). However, prenatal and postnatal ratios of embryo and pup brain DTG concentrations to mother’s plasma DTG levels were similar, 0.024 at GD 16.5 and 0.038 at PND 4. Furthermore, ratio of embryo brain DTG levels to placenta DTG levels at GD 16.5 was 0.18. In total, PK and BD data confirmed that developing embryo brain is exposed to DTG during pregnancy.

### DTG Exposure During Gestation Infrequently Leads to Neural Tube Defects

**Fig. 4:**
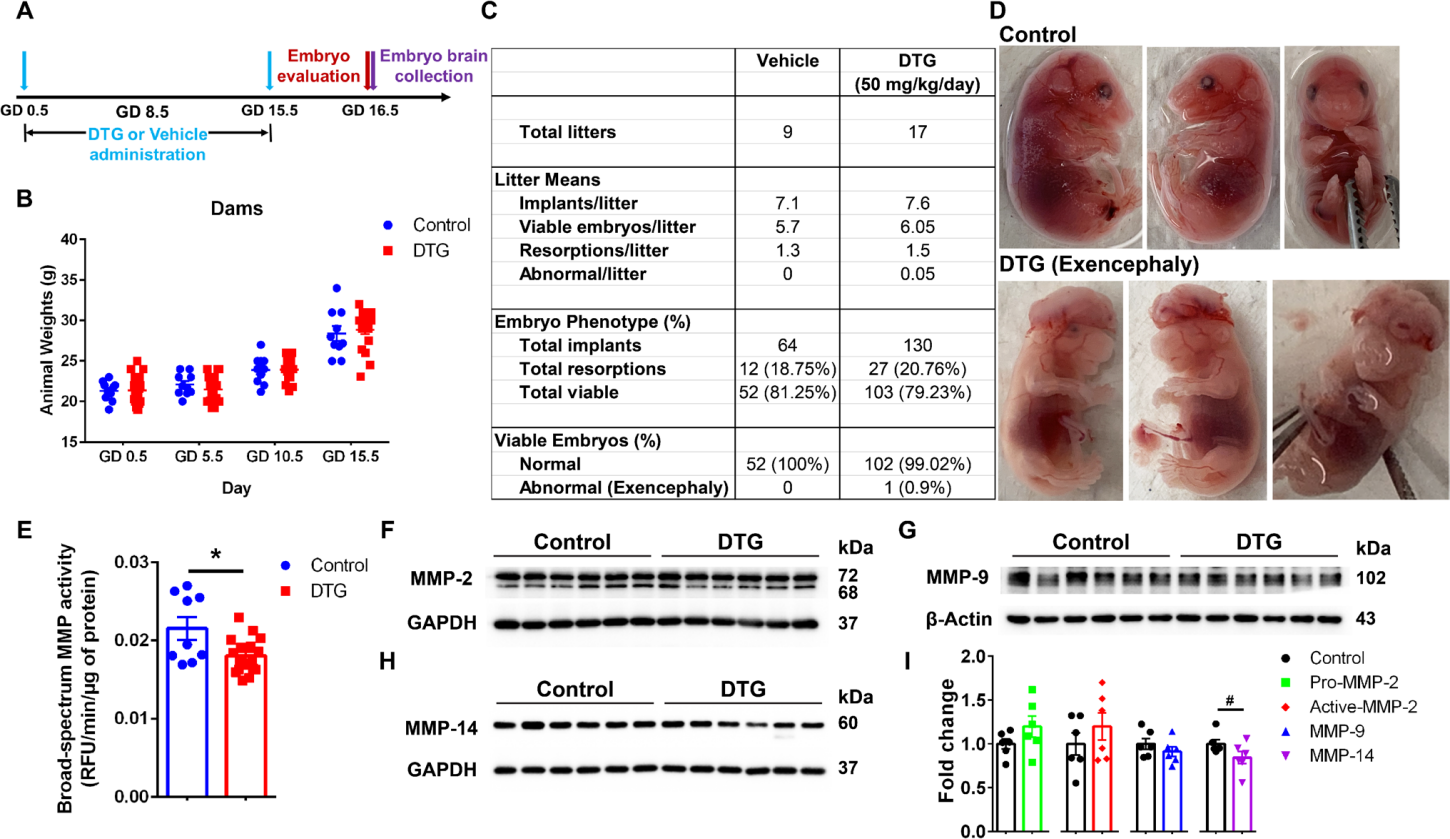
In utero DTG exposure effects on MMP activity in the fetal CNS. **A** Schematic presentation of timeline of the experiment. **B** Weights of dams from GD 0.5 to GD 15.5. No differences between DTG and control groups were observed. **C** Embryo phenotype assessment. **D** Representative images of embryos after vehicle (control) or DTG treatment at GD 16.5. From DTG group, one embryo out of 103 viable embryos showed exencephaly. **E** Fluorometric substrate assay. Broad-spectrum MMP activity in a whole brain tissue homogenate of embryos at GD 16.5 was evaluated. Data are expressed as the mean ± SEM, *N* = 9 animals (control) and *N* = 17 animals (DTG). *t* test (two-tailed) with Welch’s correction was used to compare the broad-spectrum MMP activity between DTG and control groups (*p < 0.05). **F** Protein levels of MMP-2 in embryo brain tissue. **G** Protein levels of MMP-9 embryo brain tissue. **H** Protein levels of MMP-14 embryo brain tissue. **F**, **G** Each lane is representative of six animals from each group. **I** Quantitation of protein levels in DTG compared to control using ImageJ software. Data are expressed as the mean ± SEM, *N* = 6 animals/group. *t* test (two-tailed) with Welch’s correction was used to compare protein levels of individual MMP between DTG and control (^#^*p* < 0.1)

To determine the effect of DTG during pregnancy on embryo development, female C3H/HeJ mice were administered DTG (50 mg/kg) or vehicle (control) orally every day. Daily DTG administration at a 50 mg/kg dose provided plasma drug levels in pregnant dams of 10 × the maximal DTG concentration (*C*_max_) achieved in human plasma at therapeutic doses (Fig. 3B) [43–46]. Pregnant dams were randomly assigned to DTG or vehicle group. DTG or vehicle was administered by oral gavage from GD 0.5 to GD 15.5 (Fig. 4A). Periconceptional usage of DTG by women had more adverse pregnancy outcomes compared to drug initiation during pregnancy [9, 10, 47]. Thus, DTG administration was started at the day of vaginal plug detection (GD 0.5). This approach maintained parallel drug exposures among all pregnant dams. No differences were observed in maternal body weight gain during gestation between DTG and control groups (Fig. 4B). At GD 16.5, embryos were harvested and evaluated for neural tube defects (NTDs). Embryo phenotypes in DTG or vehicle treatment groups are summarized in Fig. 4C. The litter size and viability and resorption rates were similar between groups. The mean litter size in control group was 7.1 and in DTG group was 7.6. The total resorption (%) in control dams was 18.75% (*n* = 9 litters, 12 out of 64 total implants), and in DTG-treated dams was 20.76% (*n* = 17 litters, 27 out of 130 total implants). In vehicle-treated control group, out of 52 viable embryos, no NTDs were detected (0%) (Fig. 4B, D), whereas in DTG-treated group, 1 out of 103 viable embryos showed NTDs, giving an incidence rate of 0.9% (Fig. 4C, D). The one embryo with NTD exhibited exencephaly (Fig. 4D). From total viable embryos, 99.02% (102 out of 103 viable embryos) had no abnormalities following DTG (50 mg/kg/day) treatment. Taken together, in utero DTG exposures showed limited NTDs in fetuses.

### In UteroDTG Exposure Inhibits Fetal Brain MMP Activity

To determine the effects of in utero DTG exposure on MMP activity in the developing embryo brain, randomly selected pregnant dams (C3H/HeJ mice) were administered DTG (50 mg/kg) or vehicle (control) orally each day by oral gavage from GD 0.5 to 15.5. In order to maintain periconceptional DTG usage [9, 10, 47] and equal drug exposures among dams, DTG administration was started at the day of vaginal plug detection (GD 0.5). Whole brain tissues were isolated from normal viable embryos (GD 16.5) from both groups (DTG and control) and were further processed to determine effects of DTG on MMP activity in the developing CNS. Herein, broad-spectrum MMP activity was determined from total protein isolate of brain tissue using fluorometric substrate. As DTG is a broad-spectrum MMPs inhibitor, and there are complexities in MMPs expressions, overlapping functions, and co-dependent activations/functions [20, 22, 24, 39], broad-spectrum activity was determined to reflect MMPs activities [48]. Broad-spectrum MMP activity was significantly inhibited by DTG in the embryonic brain compared to controls (Fig. 4E). Later, protein expression levels of known MMPs involved in embryonic brain development were determined. These were MMP-2, 9, and 14 (Fig. 4F–H). No significant differences were observed between DTG and controls for protein levels of any of these MMPs (Fig. 4I).

### In Utero DTG Exposure Impairs Postnatal Neurodevelopment

**Fig. 5:**
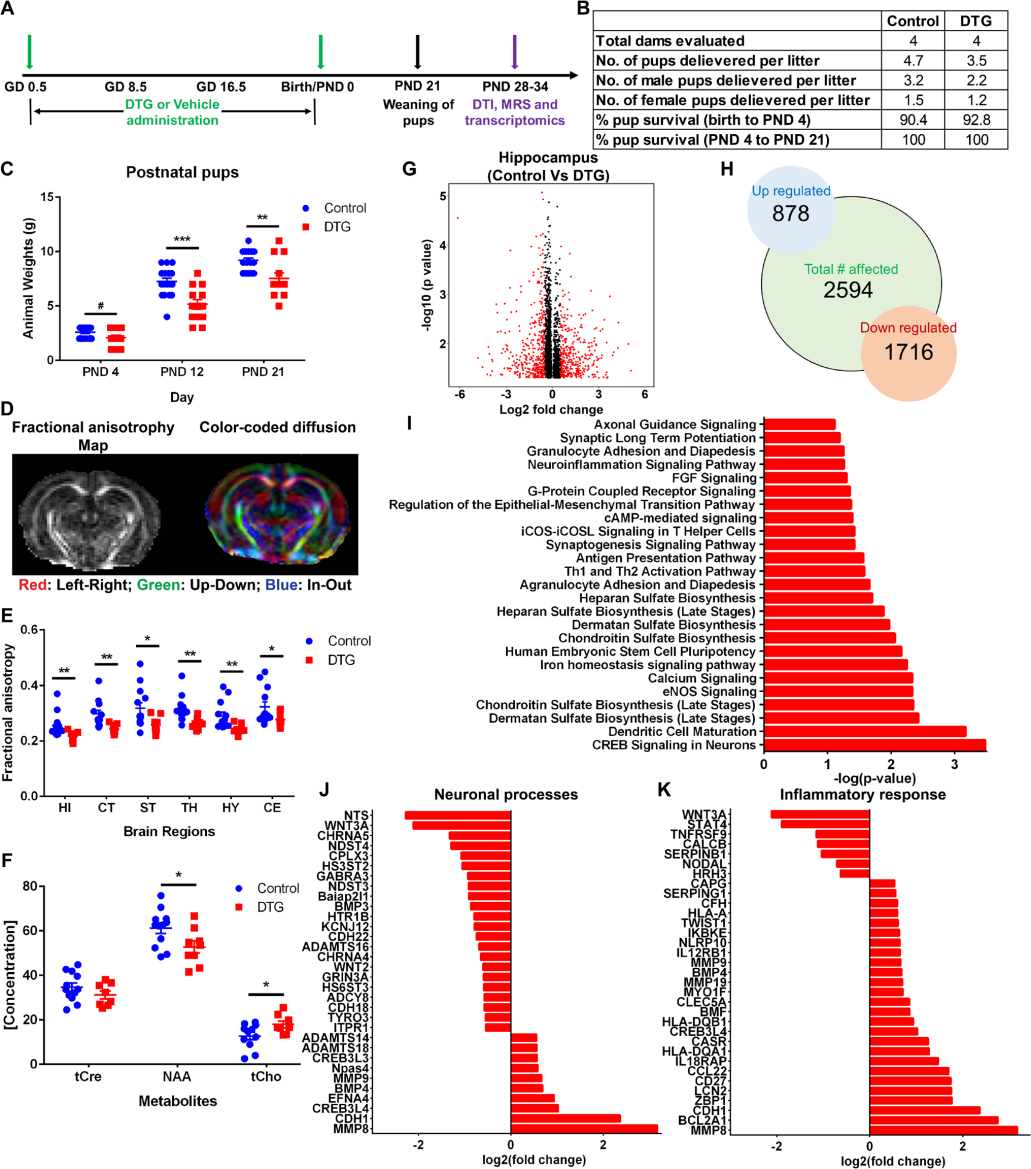
Postnatal neurodevelopment following in utero DTG exposure. **A** Schematic presentation of timeline. **B** Pregnancy and postnatal outcomes. **C** Postnatal animal (pups) weights. **D** Representative diffusion tensor imaging (DTI) image of PND 28 pup from DTG group. Red: left–right; green: up-down; blue: in–out. **E** DTI: fractional anisotropy (FA) measurements in six different brain regions: hippocampus (HI), cortex (CT), striatum (ST), thalamus (TH), hypothalamus (HY), and cerebellum (CE). **F** Magnetic resonance spectroscopy (^1^H MRS): metabolite concentration measurements in hippocampus. Total creatine (tCre), *N*-acetylaspartate (NAA), and total choline (tCho) were measured. **C**, **E**, **F** Data are expressed as mean ± SEM, *N* = minimum 8 animals/group for each parameter evaluation. Student’s *t* test (two-tailed) was used to compare animal weights at each time point, FA (DTI) in each brain region or each metabolite concentration (^1^H MRS), between DTG and control (^#^*p* < 0.1, *p < 0.05, **p < 0.01, ***p < 0.001). **G**–**K** Comparative transcriptomic analysis of total RNA from hippocampus region between DTG and control groups. *N* = 4 animals/group. **G** Volcano plot; *p* ≤ 0.05; red color: log2FoldChange ≥ 0.5 or ≤  − 0.5. **H** Total number of upregulated and downregulated genes. **I** Major affected pathways were determined by using Ingenuity pathway analysis (IPA). **J**, **K** Differentially expressed genes associated with neuronal processes and neuroinflammation

To assess the effects of in utero DTG exposure on postnatal neurodevelopment, adolescent mice pups were evaluated at PND 28 (Fig. 5A). Mice pups at this stage of development (PND 28) are equivalent to humans of 2 years of age [49]. Female C3H/HeJ mice were treated orally every day with DTG (50 mg/kg) or vehicle (control) through the entire period of gestation starting at GD 0.5 (Fig. 5A). Pregnant dams were randomly assigned to DTG or vehicle group. Average littler size was similar between DTG (3.5) and control (4.7) groups (Fig. 5B). In addition, male to female ratios per litter were similar for control (2.1) and DTG (2.0) (Fig. 5B). There were no structural malformations or variations in newborn pups in either treatment group. During the preweaning growth period, the survival rate of pups was comparable between DTG and control groups. Survival rate of pups between PND 0 and 4 was 90.4% for controls and 92.8% for DTG. Survival rate of pups was 100% between PND 4 and 21 in both groups. Further body weights of pups were measured during preweaning duration. Animal weights from DTG-treated group were significantly lower compared to control at PND 12 and 21 (Fig. 5C). Average body weights of control and DTG pups were 7.2 ± 0.2 and 9.2 ± 0.2 g (control) and 5.1 ± 0.4 and 7.5 ± 0.4 g (DTG) at PND 12 and 21, respectively. Furthermore, between age PND 28 and 34, live animals were evaluated for biochemical changes in the brain by magnetic resonance imaging (MRI) (Fig. 5D–F). All the tests were performed around 6 days post weaning to avoid confounding effect of stress related to separation from mother [50, 51]. Microstructural and metabolite measurements were conducted in live pups using bioimaging methods, diffusion tensor imaging (DTI), and magnetic resonance spectroscopy (^1^H MRS), respectively [30] (Fig. 5D–F). First, pups were scanned using DTI to determine microstructural changes in six different brain regions, hippocampus (HI), cortex (CT), striatum (ST), thalamus (TH), hypothalamus (HY), and cerebellum (CE). Color-coded diffusion directions in brain of DTG group pups are shown in Fig. 5D. In all six brain regions, fractional anisotropy (FA) values were significantly lower in the DTG group compared to those in the control group (Fig. 5E). As FA represents anisotropic movement of water molecules along the axons, reduction in FA values indicated axonal and/or myelin damage [30, 52] in developing brain of adolescent pups in the DTG-treated group. In addition, the analysis of FA values assessed the effect of in utero DTG exposures on male and female pups. This allowed evaluation of sex as a biological variant (Supplementary Fig. 4). In male pups, FA values were significantly reduced in HI, CT, ST, and HY, and trend of reduced FA values was observed in TH and CE. In female pups, significant reduction was noted only in TH and trend of decrease in HI (Supplementary Fig. 4). Further, the same pups were scanned using ^1^H MRS to measure *N*-acetylaspartate (NAA), total choline (tCho), and total creatine (tCre) in hippocampus (HI) only (Fig. 5F). NAA is a highly concentrated metabolite in the CNS and is a neuron-specific metabolite that acts as a non-invasive ^1^H MRS marker for neuronal integrity, health, and cell number [30, 53]. NAA levels in HI were significantly reduced in the DTG group compared to those in the control group (Fig. 5F), indicating neuronal and/or synaptic damage. In addition, significant increase in tCho levels in DTG group identified the neuroinflammation [54]. No differences were observed in the internal reference tCre [55]. Similar to DTI, sex differences were evaluated in the ^1^H MRS study (Supplementary Fig. 5). NAA was significantly reduced in females of DTG group compared that of control group. No significant differences between groups were observed for other metabolite concentrations in either of the sex (Supplementary Fig. 5). Together, both in vivo MRI assessments, DTI and ^1^H MRS, identified neuronal damage and neuroinflammation in pups who were exposed to DTG in utero.

For biological validation, transcriptomic evaluation of total RNA was performed on isolated HI tissue from pups following MRI assessments (Fig. 5G–K). Isolated total RNAs from HI region of brain of 4 randomly selected pups from 4 different dams for each group were used. Comparison of genes between DTG-treated group and vehicle-treated group (control) showed a total of 2594 genes significantly differentially expressed (Fig. 5G, H), out of which 1716 genes were downregulated and 878 were upregulated. Transcriptomic analysis revealed that majority of differentially expressed genes were associated with pathological processes/pathways including aberrant biosynthesis of extracellular matrix components, neuroinflammation, axonal guidance and synaptogenesis, and infiltration of immunocytes in HI of pups from DTG group compared to controls (Fig. 5I–K). Major affected genes which showed aberrant biosynthesis of extracellular matrix components were NDST3, NDST4, HS3ST2, and HS6ST3 (Fig. 5I–K). In addition, differentially expressed genes served to confirm putative mechanisms for neuronal damage, inflammation, and immune cell infiltration (Fig. 5I–K). Genes associated with these mechanisms are categorized by differential expression. Those with increased expression were identified as MMP-8, MMP-9, MMP-19, CDH1, CREB3L4, CREB3L3, EFNA4, BMP4, Npas4, ADAMTS18, ADAMTS14, BCL2A1, ZBP1, LCN2, CD27, CCL22, IL18RAP, HLA-DQA1, CASR, IL12RB1, NLRP10, HLA-DQB1, BMF, CLEC5A, MYO1F, IKBKE, TWIST1, HLA-A, CFH, SERPING1, and CAPG (Fig. 5J, K). Those with decreased expression were ITPR1, TYRO3, CDH18, ADCY8, HS6ST3, GRIN3A, WNT2, CHRNA4, ADAMTS16, CDH22, KCNJ12, HTR1B, BMP3, Baiap2l1, NDST3, GABRA3, HS3ST2, CPLX3, NDST4, CHRNA5, WNT3A, NTS, HRH3, NODAL, SERPINB1, CALCB, TNFRSF9, and STAT4 (Fig. 5J, K). Moreover, top network identified TNF-α as a master regulator with 17 focus molecules, indicating the inflammatory response (Supplementary Fig. 6). According to the drug ratio relationship between dam’s plasma and embryo brain (Fig. 3), DTG level in pup’s brain is predicted to be below detection limit (0.1 ng/g) at PND 28 after cessation of DTG administration to dam at the day of birth of pups. Thus, inhibition of MMP activity due to direct drug interaction is not expected at PND 28 or thereafter in the current study design (Fig. 5A). In all, the observed postnatal neuropathology can be linked to DTG’s effect on the developing embryo CNS.

## Discussion

With the widespread global use of DTG-based regimens and noted ADE in clinical and preclinical studies, defining in utero effects of drug on fetal development is timely. This includes the mechanism(s) of action on the developing CNS [2, 9–13]. Prior works focused on determining relationships between folate levels or transport pathways and DTG-associated birth defects failed to conclusively generate any cause and effect relationships [11, 56–59]. Exploring alternative mechanisms was required [11, 56–59]. Herein, for the first time, we show that DTG inhibits MMPs activities in fetal brain during gestation and as such impairs neurodevelopment. DTG was found to be a broad-spectrum inhibitor of MMPs. The drug was found to bind Zn^++^ at the catalytic domain, leading to inhibition of MMPs activities. Moreover, studies in pregnant mice showed that DTG can cross the placental barrier, accumulate in the fetal CNS, and inhibit MMP activity during the critical period of brain development. Further postnatal evaluation of brain health following in utero DTG exposure showed neuronal impairment associated with neuroinflammation.

The role of MMPs in neural development is of critical importance as dysregulation of MMP activity is a hallmark of neurodevelopmental disorders [15–26]. MMPs regulate multiple processes of neurodevelopment. These include, but are not limited to, angiogenesis, neurovasculature remodeling, establishment and integrity of blood–brain barrier (BBB), neurogenesis, neuronal migration, myelination, axonal guidance, synaptogenesis, synaptic plasticity, and tissue remodeling [15–26]. Several members of MMP family have been implicated in such processes. Notably, four MMPs, MMP-2, 8, 9, and 14, have been studied extensively for their roles in angiogenesis and neurogenesis [15–26]. Our data showed that DTG is a broad-spectrum MMP inhibitor with strong inhibitory effect (lowest IC_50_ values among tested MMPs), particularly on MMP-2, 8, 9, and 14. In addition, clinical trials have revealed that prolonged treatment with broad-spectrum MMP inhibitors has severe adverse effects on physiological processes [39]. Thus, being a broad-spectrum inhibitor of MMPs and being able to reach to embryonic CNS during critical period of development, DTG imposes a noted risk for neurodevelopment.

In addition to lower body weights, neuronal damage and neuroinflammation was observed in adolescence mouse pups (PND 28–34) of DTG-treated group during postnatal growth assessments. It is noteworthy that DTG administration to mother was stopped at birth of pups and DTG concentrations in brain tissue were very low or undetectable level at PND 28. These data suggest that the aberrations resulted from inhibition of MMPs activities during gestation were not recovered or extended to observed pathology up to PND 28. Interestingly, increased RNA levels of MMP-8, MMP-9, and MMP-19 were observed in HI at postnatal transcriptomic evaluation. DTI, ^1^H MRS, and transcriptomic data validated neuronal injury, neuroinflammation, and immunocyte infiltration into the brain, which could explain increase in these MMPs [60]. In our prior studies, we examined metabolomic profiles in adult rodent brain tissues that were affected by DTG. These works included analyses of energy and oxidative stress pathways [61]. Interestingly, the oxidative stress pathways induced by DTG were significantly attenuated when this antiretroviral drug was administered as an intramuscular extended-release poloxamer-coated aqueous nanoformulation. These therapeutic benefits of nanoformulations were attributed to slow release of the parent drug from the depot over an extended period of time and neuroprotective properties of poloxamers. Thus, such neuroprotective activities seen by formulation design demonstrate the potential of extended-release nanoformulations to attenuate DTG-associated neurotoxicity that includes the findings described in this report.

The chemical property of DTG to chelate divalent cations enables it to interact with Zn^++^ in the catalytic domain of MMPs. This results in the inhibition of MMPs activities. All INSTIs including CAB, BIC, RAL, and elvitegravir (EVG) possess MBP to interact with metal ions. Our preliminary molecular docking evaluation showed that CAB, BIC, and RAL can interact with Zn^++^ at the catalytic domain of MMP-2 and MMP-14, indicating that not only DTG but the entire INSTI drug class could affect MMPs activities. Further research efforts are required to evaluate the effects of other INSTIs on MMPs and on CNS development following in utero exposure. With long-acting CABENUVA (CAB co-administered with rilpivirine) approved recently (January 2021) by the US FDA as a monthly injectable [62], and with positive data of once in two months CAB-regimen long-acting injectable from phase III clinical trial (ATLAS-2 M study) [63], effects of sustained levels of CAB on MMPs remain to be determined.

Identifying the roles of the MMPs and their broad-spectrum inhibition in physiological processes is complex. Indeed, clinical trials with broad-spectrum MMP inhibitors revealed that prolonged treatment causes ADE. This reflected in cessation of human clinical trials for more than fifty broad-spectrum MMP inhibitors [39]. As DTG is a zinc-binding inhibitor, observed undesired adverse effects on the developing CNS were mostly due to broad-spectrum MMP inhibition. However, potential cross-inhibition of a disintegrin and metalloproteinase (ADAM) family members and of a disintegrin and metalloproteinase with thrombospondin motifs (ADAMTS) family members by DTG, in addition to inhibition of MMP activity, could be yet another potential mechanism [39, 64, 65]. Such cross-inhibition of ADAM or ADAMTS members by zinc-binding broad-spectrum MMP inhibitors is known due to structural similarities between MMPs and ADAM/ADAMTS at catalytic domains. DTG is a potent INSTI with IC_50_ value of 2.7 nM [66]. However, the recorded IC_50_ values for DTG inhibition of MMP activities were high. These values were ranged between 10 and 300 µM. Moreover, calculated IC_50_ values for DTG, in the current study, are higher than clinically relevant concentrations after considerations for required plasma-protein binding adjustments [59]. Long-term, daily administration of DTG in HIV-1-infected persons, transfer easily across the placental barrier, and exposure of a developing CNS to drug during the entire gestational period and through breast feeding require further evaluation. Certainly, observed DTG-associated MMP inhibition was statistically significant; however, inhibition can be realized at modest extent. It is well known that balanced regulation of MMPs activities is essential for normal biological functioning and growth [19, 20, 39]. Dysregulation of activity of any single or multiple MMPs in either direction, increase or decrease, leads to adverse effects. Fetal neurodevelopmental processes during gestation are highly susceptible to any chemical challenges. Thus, DTG readily existing at embryo CNS throughout the gestational period and reduced MMPs activities at notable extent can potentially cause damage and lead to developmental disorders. It, also, needs to be noted that evaluation of MMP activity in embryo CNS was completed only at GD 16.5. Other gestational time points and intensity of the drug impact at those time points remain unknown. However, observed postnatal brain aberrations in adolescent mice pups affirmed DTG-associated fetal brain toxicity as consequence of in utero exposures. Also, it needs to be emphasized that the current study was designed for proof-of-concept only. The intent was to determine any or all effect of in utero DTG exposure on MMP activity in embryo brain and whether or not it could affect prenatal and or postnatal neurodevelopment. Thus, for this initial assessment, a higher dosage (50 mg/kg/day of mice weight) was selected that could achieve 10 × the maximal DTG concentration (*C*max) recorded in human plasma. Recently, increased rates of fetal defects in mice embryos exposed to therapeutic DTG doses during pregnancy compared to supratherapeutic levels were reported [11]. The results of these findings supported a causal relationship of DTG at therapeutic doses with increased risk for fetal defects. This included neural tube defects (NTDs). Assessments of the findings recorded at a clinical therapeutic dosage will be the focus of our own future works.

The percentage of NTDs following in utero DTG exposure was low and seen only in 1 out of 103 embryos (0.9%). Although the number of litters evaluated were few (17 total) in the current study, the observation is similar to overall clinical prevalence of NTDs among infants born to women receiving DTG at conception (~ 0.3%) [10, 67]. In addition to adverse pregnancy outcomes, DTG is associated with neuropsychiatric adverse events (NPAEs) in adults [68, 69] and clinically significant weight gain, especially in females [70–73]. Notably, NPAEs are usually reversible upon discontinuation of DTG. Inhibition of certain MMP’s activity, related compensatory increase in other MMP activity and/or expression and affected MMP-dependent upstream or downstream biological and immunological processes, could be an underlying mechanism for these known DTG-associated adverse effects [23, 39, 74–78]. Recently, vascular defects (cranial and spinal and hemorrhagic bleeds) were reported at 2- to threefold higher rate in mice embryos exposed to DTG in utero. In these studies, vascular defects were observed at therapeutic levels of DTG [11], supporting the notion that DTG could affect vascular integrity during embryo development [11]. Angiogenesis and vascular remodeling are dependent, in part, on MMP activity [79, 80]. Synthetic MMPs inhibitors and endogenous tissue inhibitors of metalloproteinases (TIMPs) possess antiangiogenic properties [79, 80]. Moreover, MMP knockout mice show angiogenesis defects and impaired vascular remodeling [19, 20]. Thus, DTG inhibition of MMPs activities could, in measure, explain such vascular defects observed in the mouse embryo [11]. Potential dose-escalation DTG effects on vascular development and MMPs activities require future investigation.

In coming years, significant numbers of fetuses will be exposed to DTG. Due to a breakthrough pricing agreement made in September 2017 to accelerate the availability of generic DTG-based regimens in RLCs at the cost of $75 US dollars/person/year, around 100 RLCs have implemented transitioning to DTG-based regimen in national treatment guidelines by mid-2020 [81]. In 2019, ~ 6.9 million people had access to generic DTG regimen and up to 15 million people worldwide would be treated with DTG by the year 2025 [7, 8, 81, 82]. This includes women of child-bearing age who remain a significant infected population (UNAIDS data, 2020) [83]. It is estimated that there are 15.5 million women, 15–49 years old, living with HIV worldwide [84]. Moreover, women accounted for about 48% of all new HIV-1 infections in 2019 [83]. Thus, with increased availability of DTG-based regimens and its inclusion in recommended national treatment guidelines globally, significant population of women of child-bearing age will be treated with DTG-based regimens.

Although the current proof-of-concept study provides a novel mechanism cross-validated with multidisciplinary research approach, a few limitations are recognized. First, dose-dependent cause and effect assessment was not performed. The study was conducted at one dosage (50 mg/kg), which achieved supratherapeutic plasma DTG levels in pregnant dams. Second, embryos were evaluated only at one time point of gestation (GD 16.5). Third, the study was focused on brain development; however, the effects on different developmental processes that are dependent on MMP activity such as neurogenesis and angiogenesis were not evaluated. Fourth, the study did not include assessment of functional and structural anomalies in other developmental organs such as placental, cardiac, or skeletal defects. Comprehensive evaluations consisting effect of dose-dependent DTG inhibition of MMPs activities on neurodevelopment that would include neurogenesis and angiogenesis during different stages of gestation will be completed in future studies. This includes any links to long-term postnatal neurologic outcomes.

The mechanisms that affect human neurodevelopment following in utero exposure of DTG are not known. An observational clinical study reported increased risks of neurodevelopmental aberrations by in utero DTG exposure in HIV-exposed but uninfected (CHEU) children [2]. In full consideration of prior human studies, it is timely to elucidate any potential links to adverse events, no matter how infrequent, in order to provide the most effective care to women and their fetuses at risk or infected by HIV-1. Continued monitoring of children during development will determine accurate DTG-linked neurodevelopmental effects with MMP activities. These studies would be completed during pregnancy and postnatal development. Further understanding for drug formulation and delivery would serve to maximize the drug’s benefits and minimize any untoward effects. In this report, we demonstrate that DTG inhibition of MMPs activities during gestation can affect pre- and postnatal neurodevelopment in mice. The study provides a new potential mechanism that can serve to advance translational research in evaluating the effects of in utero exposure to DTG and potentially to other new HIV-1 integrase inhibitors on neurodevelopment.

## Supplementary Information

Below is the link to the electronic supplementary material.

Supplementary file1 (PDF 1611 KB)

## Data Availability

The datasets used during the current study are available from the corresponding author on reasonable request.
