# Supplementary material for: Dolutegravir Inhibition of Matrix Metalloproteinases Affects Mouse Neurodevelopment
Source: Mol Neurobiol. 2021 Aug 14;58(11):5703–21. doi: 10.1007/s12035-021-02508-5 (PMC8599359; doi:10.1007/s12035-021-02508-5)
Supplement: Supplementary file 1 — Supplementary file1 (PDF 1611 KB) [file 12035_2021_2508_MOESM1_ESM.pdf]

Journal name: Molecular Neurobiology

Dolutegravir Inhibition of Matrix Metalloproteinases Affects Mouse Neurodevelopment

Aditya N. Bade[1]*, JoEllyn M. McMillan[1], Yutong Liu[1,2],
Benson J. Edagwa[1], Howard E. Gendelman[1,3]*

[1]Department of Pharmacology and Experimental Neuroscience, University of Nebraska Medical Center, Omaha, NE 68198, USA.
[2]Department of Radiology, University of Nebraska Medical Center, Omaha, NE 68198, USA.
[3]Department of Pharmaceutical Sciences, University of Nebraska Medical Center, Omaha, NE 68198, USA.

***Corresponding author:**

Aditya N. Bade, Ph.D., Department of Pharmacology and Experimental Neuroscience, University of Nebraska Medical Center, Omaha, NE 68198-5800, USA; phone: 402-559-8916; fax: 402-559-7495; email: aditya.bade@unmc.edu, ORCID: https://orcid.org/0000-0003-2511-4461 (for correspondence);

Howard E. Gendelman, M.D., Department of Pharmacology and Experimental Neuroscience, University of Nebraska Medical Center, Omaha, NE 68198-5880, USA; phone: 402-559-1173; fax: 402-559-3744; email: hegendel@unmc.edu, ORCID: https://orcid.org/0000-0002-7831-0370 (for submission, contact and correspondence)

Supplementary Information

Supplementary Figures

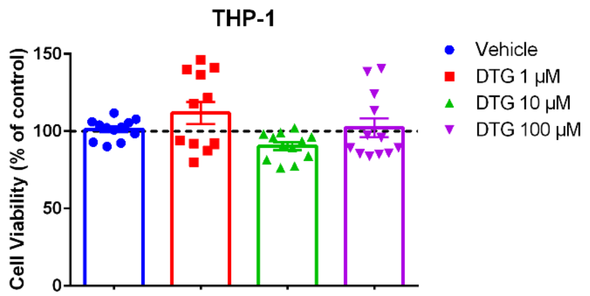

Supplementary Figure 1. Cytotoxicity assay. The cellular viability in THP-1 cells was determined after 18 hours of DTG treatment over a range (1-100 µM) of concentrations. Cytotoxicity was not observed at any concentration. Data are expressed as the mean ± SEM for N = 12 biological replicates.

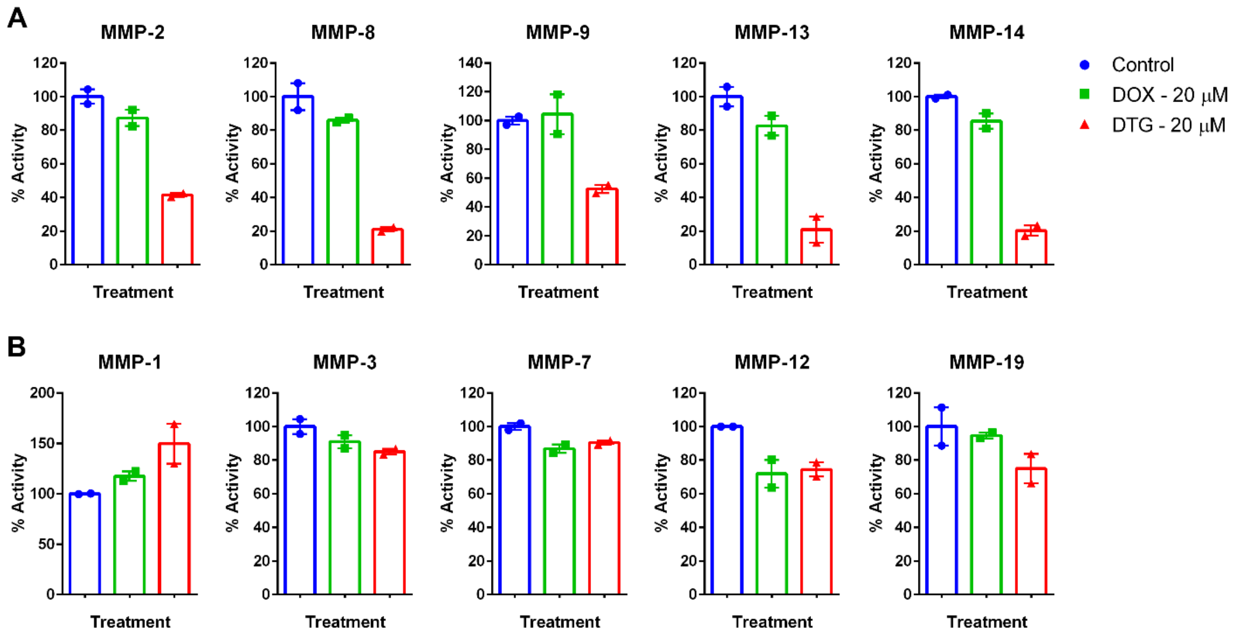

Supplementary Figure 2. MMP inhibition activity. (**A** and **B**) MMP Inhibition profile of DTG was validated in comparison to doxycycline (DOX, only US FDA approved broad spectrum MMP inhibitor) using fluorometric substrate assay. A panel of ten human recombinant MMP enzymes were used. Data are expressed as the mean ± SEM, N = 2 biological replicates

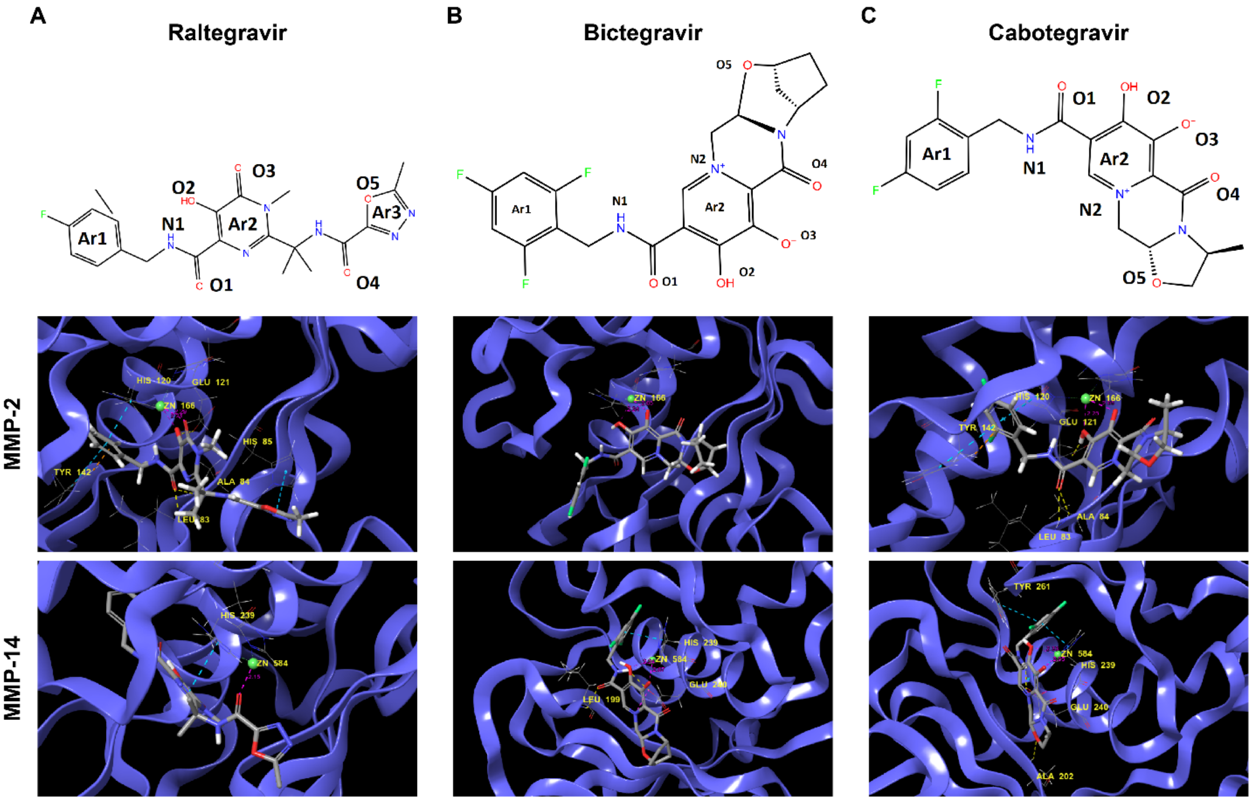

Structure	Energy (kcal/mol)
MMP-2	-6.29
MMP-14	-8.66

Structure	Energy (kcal/mol)
MMP-2	-10.97
MMP-14	-14.53

Structure	Energy (kcal/mol)
MMP-2	-8.5
MMP-14	-12.98

MMP-2 Interactions			
Ligand	Receptor	Type	Distance (Å)
Ar1	His 120	Pi stacking	3.47
Ar1	Tyr 142	Pi stacking	5.16
Ar1	Zn 166	Cation pi	5.19
O1	Leu 83	Hydrogen bond	1.78
O1	Ala 84	Hydrogen bond	2.14
O2	Glu 121	Hydrogen bond	2.16
O2	Zn 166	Metal coordinatior	2.21
O3	Zn 166	Metal coordinatior	2.29
Ar3	His 85	Pi stacking	5.28

MMP-14 Interactions			
Ligand	Receptor	Type	Distance (Å)
Ar2	His 239	Pi stacking	3.63
Ar2	Zn 584	Cation pi	4.98
O4	Zn 584	Metal coordinatior	2.15

MMP2 Interactions			
Ligand	Receptor	Type	Distance (Å)
O2	Zn 166	Metal coordinatior	2.24
O3	Zn 166	Metal coordinatior	2.05
O3	Zn 166	Salt bridge	2.05

MMP14 Interactions			
Ligand	Receptor	Type	Distance (Å)
Ar1	His 239	Pi stacking	3.69
O1	Leu 199	Hydrogen bond	2.61
O2	Zn 584	Metal coordinatior	2.24
O3	Zn 584	Metal coordinatior	2.07
O3	Zn 584	Salt bridge	2.07
N1	Glu 240	Hydrogen bond	2.64
N2	Glu 240	Salt bridge	3.61

MMP2 Interactions			
Ligand	Receptor	Type	Distance (Å)
Ar1	His 120	Pi stacking	3.34
Ar1	Tyr 142	Pi stacking	5.07
O1	Leu 83	Hydrogen bond	1.99
O1	Ala 84	Hydrogen bond	2.4
O2	Glu 121	Hydrogen bond	1.92
O2	Zn 166	Metal coordinatior	2.25
O3	Zn 166	Metal coordinatior	2.05
O3	Zn 166	Salt bridge	2.05

MMP14 Interactions			
Ligand	Receptor	Type	Distance (Å)
Ar1	His 239	Pi stacking	3.76
Ar1	Tyr 261	Pi stacking	5.36
Ar2	His 239	Pi stacking	4.98
O2	Zn 584	Metal coordinatior	2.33
O3	Zn 584	Metal coordinatior	2.05
O3	Zn 584	Salt bridge	2.05
O5	Ala 202	Hydrogen bond	2.42
N1	Glu 240	Hydrogen bond	2.15
N2	Glu 240	Salt bridge	3.15

Supplementary Figure 3. **Molecular interaction of INSTIs with MMP.** Molecular interactions of (**A**) raltegravir (RAL), (**B**) bictegravir (BIC), and (**C**) cabotegravir (CAB) with MMP-2 and MMP-14 were evaluated. (**A – C**) Chemical structures of RAL, BIC and CAB with ligand labelling used for molecular docking are shown. 3D representations of molecular docking complex of each INSTI (RAL, BIC or CAB) with MMP-2 and MMP-14 are shown. 3D representations of molecular docking complexes are provided in ribbon (blue color) format. Calculated binding energies for each MMP using Schrodinger's software suite are provided in tabular format. Interactions with Zn++ (green ball) and other amino acids (yellow color) are identified. Interaction with Zn++ are shown by pink

dotted line. Hydrogen bond interaction with amino acids is shown by yellow dotted line. Pi staking is shown by blue dotted line. Interaction details for each drug-protein complex are provided in the tabular format.

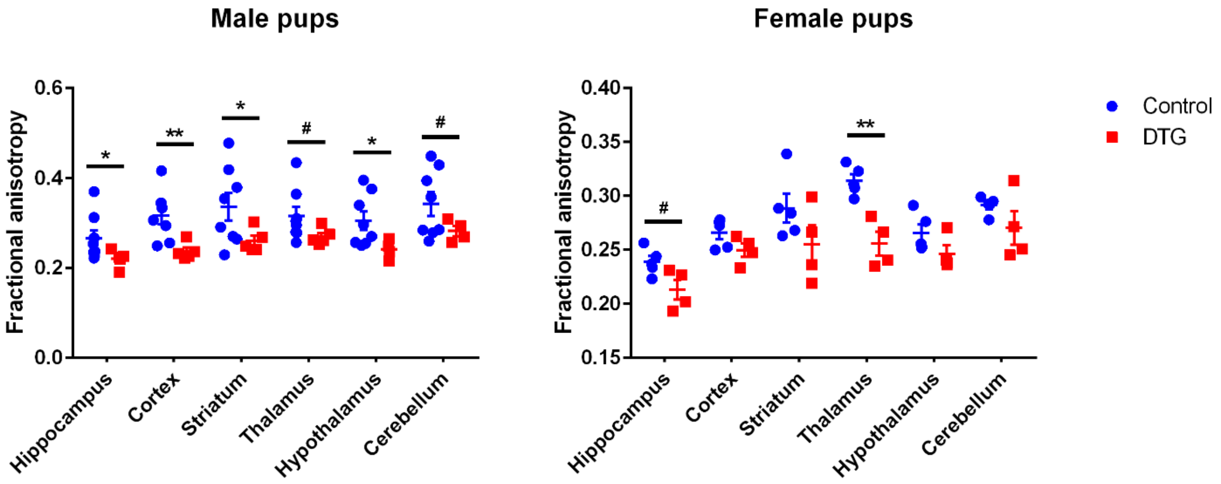

Supplementary Figure 4. Diffusion tensor imaging (DTI). Fractional anisotropy (FA) values were compared between male and female pups. FA values were measured in six different brain regions, hippocampus (HI), cortex (CT), striatum (ST), thalamus (TH), hypothalamus (HY), and cerebellum (CE). Data included in the Figure 5E were further separated according to sex of an animal to determine biological variance. Data are expressed as mean ± SEM, N = minimum 4 animals/group. Student's t test (two-tailed) was used to compare FA (DTI) at each brain region between DTG and control. ($^{\#}P < 0.1$, $^{*}P < 0.05$, $^{**}P < 0.01$).

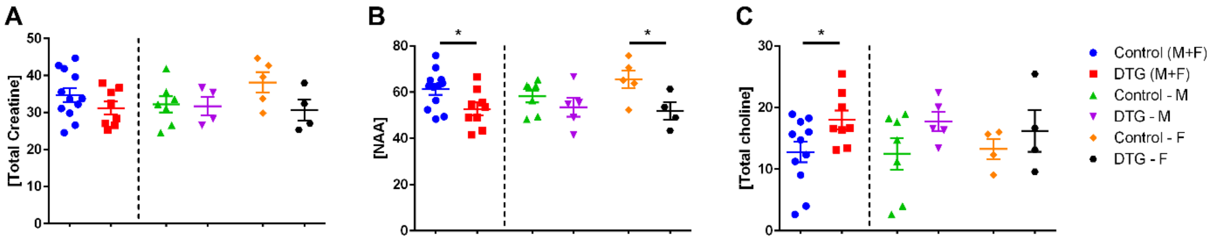

Supplementary Figure 5. Magnetic resonance spectroscopy (^{1}H MRS). Metabolite concentrations were measured in hippocampus and compared between male and female pups. Concentrations of total creatine (tCre), N-acetylaspartate (NAA) and total choline (tCho) were measured. Data included in the Figure 5F were further separated according sex of an animal to determine biological variance. Data are expressed as mean ± SEM, N = minimum 4 animals/group. Student's t test (two-tailed) was used to compare individual metabolite concentrations between DTG and control. ($^{\#}P < 0.1$, $^{*}P < 0.05$). M = Male, F = Female, M+F = Male + Female.

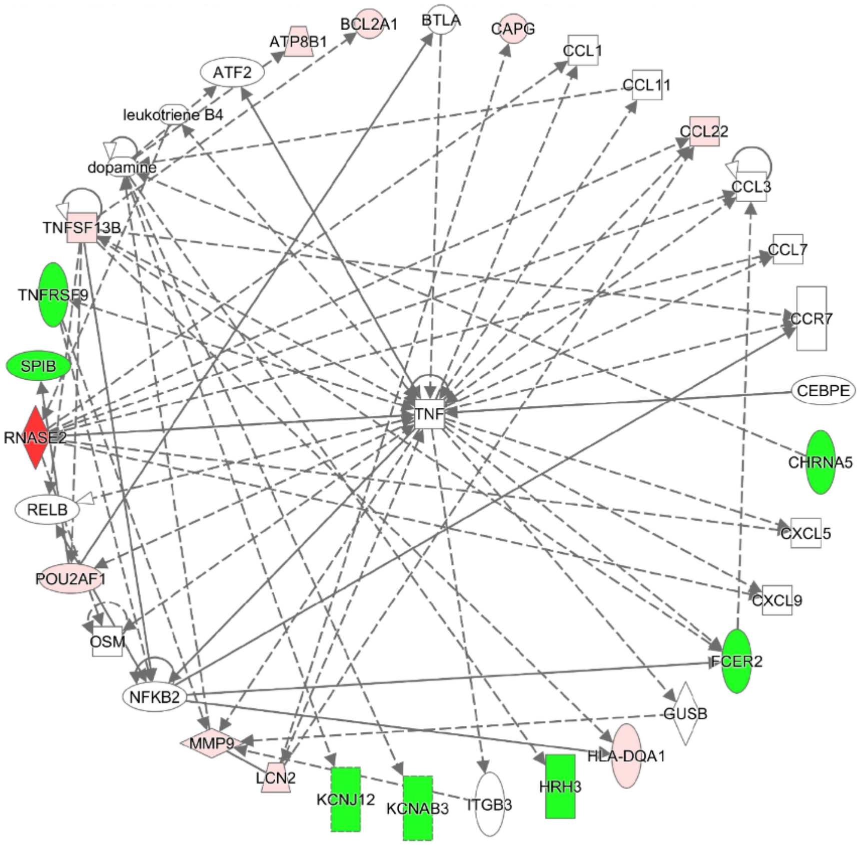

Supplementary Figure 6. Comparative transcriptomic analysis of total RNA between DTG and control groups. Gene expression differences were assessed in hippocampus tissue. Network evaluation by Ingenuity Pathway Analysis (IPA) identified top network with TNF-α as a master regulator with 17 focus molecules. Green color – increase in expression; Red (dark/light) color: decrease in expression. N = 4 animals/group.

Supplementary Methods

Reagents

DTG was purchased from BOC Sciences (Shirley, NY). Human recombinant MMP-2 and -9, proenzymes, and p-aminophenylmercuric acetate (APMA) were purchased from EMD Millipore (Burlington, MA). Coomassie Brilliant Blue R-250 and Coomassie Brilliant Blue R-250 destaining solution were purchased from BIO-RAD (Hercules, CA). Dimethyl sulfoxide (DMSO), PMA, and MTT were purchased from Sigma-Aldrich (St. Louis, MO). Gibco RPMI 1640 medium was purchased from Thermo Fisher Scientific (Waltham, MA). UltraPure Tris was purchased from Invitrogen (Carlsbad, CA). Trizma hydrochloride (Tris-HCl), gelatin from porcine skin, sodium chloride (NaCl), calcium chloride dihydrate, triton X-100, brij L23 solution, and glycerol were purchased from Sigma-Aldrich (St. Louis, MO).

PK and BD of DTG during pregnancy

DTG levels were quantitated in plasma, and tissue homogenates by ultraperformance liquid chromatography tandem mass spectrometry (UPLC-MS/MS). Briefly, for plasma DTG analysis, 1 ml of acetonitrile (ACN) and 10 µl of internal standard (IS) solution were added to 25 µl of plasma. DTG-d3 at a final concentration of 50 ng/ml served as the IS. Samples were vortexed and centrifuged at 17,000×*g* for 10 min at 4 °C. Supernatants were dried using a speed-vac and reconstituted in 100 µl 50% (v/v) ACN in water. The samples were vortexed and centrifuged at 17,000 x g at 4˚C for 10 min and the supernatant was used for DTG quantitation. For tissue analysis, 50–200 mg of tissue (placenta, fetal brain) was homogenized in 9 volumes of 90% (v/v) methanol (MeOH) in water using a Qiagen TissueLyzer II (Valencia, CA). Subsequently, 280 µl of MeOH, 10 µl of 80% (v/v) MeOH in water, and 10 µl IS were added to 100 µl of tissue homogenate. Samples were then vortexed, left for 5 min on ice and centrifuged at 17,000 × g and 4˚C for 15 min. Twenty µl of supernatant was mixed with 80 µl of 50% (v/v) ACN in water. The samples were centrifuged at 17,000 × g at 4˚C for 10 min and the supernatant was used for analysis. Standard

curves were prepared using control plasma and tissue in a similar manner as the study samples, with the addition of 10 μl of 10× concentrated DTG spiking solution to provide a final DTG concentration range of 0.2–2000 ng/ml. Chromatographic separation of 10 μl sample injections were achieved with an ACQUITY UPLC-BEH Shield RP18 column (1.7 μm, 2.1 mm × 100 mm) affixed with an ACQUITY BEH shield RP18 Vanguard column (2.1 mm × 5 mm) using a 10-min gradient of mobile phase A (7.5 mM ammonium formate in water adjusted to pH 3 using formic acid) and mobile phase B (100% ACN) at a flow rate of 0.25 mL/min. The initial mobile phase composition was 40% B for the first 3 min at which time it was increased to 86% B over 30 s and held constant for 5 min. Mobile phase B was then reset to 40% over 15 s and held for 1.25 min for equilibration. Multiple reaction monitoring (MRM) transitions for DTG and DTG-d3 were 420.075 > 277.124 and 422.841 > 129.999 *m*/*z*, respectively, were used for quantitation. All solvents for sample processing and UPLC-MS/MS analysis were LC-MS-grade (Fisher Scientific, Hampton, NH).
